# Studies of *Caenorhabditis elegans* DAF-2/insulin signaling reveal targets for pharmacological manipulation of lifespan

**DOI:** 10.1111/j.1474-9726.2006.00188.x

**Published:** 2006-02

**Authors:** Minaxi S Gami, Catherine A Wolkow

**Affiliations:** Laboratory of Neurosciences, Gerontology Research Center, National Institute on Aging, National Institutes of HealthBaltimore, MD 21224, USA

**Keywords:** Aging, *C. elegans*, FOXO, insulin, lifespan, phosphoinositol 3-kinase

## Abstract

Much excitement has arisen from the observation that decrements in insulin-like signaling can dramatically extend lifespan in the nematode, *Caenorhabditis elegans*, and fruitfly, *Drosophila melanogaster*. In addition, there are tantalizing hints that the IGF-I pathway in mice may have similar effects. In addition to dramatic effects on lifespan, invertebrate insulin-like signaling also promotes changes in stress resistance, metabolism and development. Which, if any, of the various phenotypes of insulin pathway mutants are relevant to longevity? What are the genes that function in collaboration with insulin to prolong lifespan? These questions are at the heart of current research in *C. elegans* longevity. Two main theories exist as to the mechanism behind insulin's effects on invertebrate longevity. One theory is that insulin programs metabolic parameters that prolong or reduce lifespan. The other theory is that insulin determines the cell's ability to endure oxidative stress from respiration, thereby determining the rate of aging. However, these mechanisms are not mutually exclusive and several studies seem to support a role for both. Here, we review recently published reports investigating the mechanisms behind insulin's dramatic effect on longevity. We also spotlight several *C. elegans* genes that are now known to interact with insulin signaling to determine lifespan. These insights into pathways affecting invertebrate lifespan may provide a basis for developing strategies for pharmacological manipulation of human lifespan.

## Introduction

Insulin-like signaling has dramatic effects on longevity in several organisms. Genetic mutations reducing insulin-like signaling can extend adult lifespan in *Caenorhabditis elegans*, *Drosophila melanogaster* and, possibly, mice (Tatar *et al*., 2003; Baba *et al*., 2005). An enticing extension of these findings is that insulin-like signaling will also affect human longevity. In addition to the potential for endocrine control of aging by insulin, signaling through insulin-like pathways is also a major factor in the development of cancer and diabetes, both of which have increased incidence at older ages (Saltiel & Kahn, 2001; Osaki *et al*., 2004). Cellular insulin resistance is a key feature in diabetes mellitus, a serious aging-related disease that is increasing with the growing epidemic of obesity (Vollenweider, 2003). Cancer can arise when mutations activate signaling downstream of insulin/IGF-I receptors leading to dysregulated cell growth (Cantley & Neel, 1999; Lawlor & Alessi, 2001). Thus, the components of insulin-like signaling pathways are at the center of several important aspects of aging and longevity. As such, pharmacological manipulation of this signaling pathway holds significant promise for improving human health in old age.

Here, we review recent studies that have uncovered new details about how insulin signaling affects lifespan in the nematode *C. elegans*. We examine the components of the *C. elegans* insulin pathway and several newly described interacting pathways. Finally, we will discuss the cellular effects of insulin signaling that may affect lifespan and discuss opportunities for pharmacological intervention highlighted by *C. elegans* biology.

## The pathways mediating insulin-like signaling in *C. elegans* and humans

**Fig. 1:**
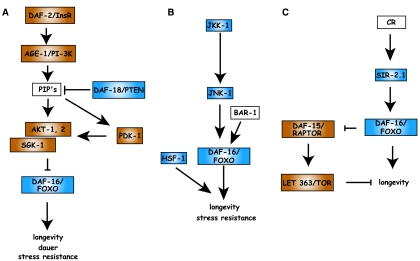
Pathways controlling longevity in collaboration with DAF-16/FOXO in *Caenorhabditis elegans*. Orange designates genes whose loss-of-function phenotype is increased lifespan while blue designates genes whose loss-of-function phenotype is reduced lifespan. (A) Ligands activate the DAF-2/InsR receptor that recruits AGE-1/PI3K to the cell membrane. AGE-1/PI3K generates phospholipid signals, PIPs, which activate serine/threonine kinases AKT-1, -2, PDK-1 and SGK-1. These kinases phosphorylate DAF-16/FOXO preventing nuclear translocation. DAF-18/PTEN negatively regulates AGE-1 signaling by dephosphorylating PIPs. (B) In response to cellular stress, JNK-1 promotes DAF-16 translocation into the nucleus, activating genes to increase stress resistance (Oh *et al*., 2005). HSF-1 is activated by stress and promotes the expression of *hsp*s in collaboration with DAF-16 (Hsu *et al*., 2003; Morley & Morimoto, 2004). BAR-1/beta-catenin acts as a cofactor for DAF-16-mediated expression of antioxidant genes and is required for wild-type lifespan (Essers *et al*., 2005). (C) Pathways that sense metabolic status and prolong lifespan. TOR is a sensor of nutrient availability that coordinates protein synthesis and metabolism in both vertebrates and invertebrates. In *C. elegans*, mutations inactivating TOR, encoded by the *let-363* gene, cause developmental arrest and can increase adult lifespan in a *daf-16*-independent manner (Vellai *et al*., 2003; Jia *et al*., 2004). *daf-15* encodes the RAPTOR subunit for TOR/LET-363 and is transcriptionally repressed by DAF-16/FOXO (Jia *et al*., 2004). Metabolic status can also be transduced by the NAD-dependent protein deacetylase, SIR-2.1. Increased *sir-2.1* activity increases lifespan in a *daf-16*-dependent manner, and DAF-16 may be a substrate for SIR-2.1 deacetylation (Tissenbaum & Guarente, 2001).

**Fig. 2:**
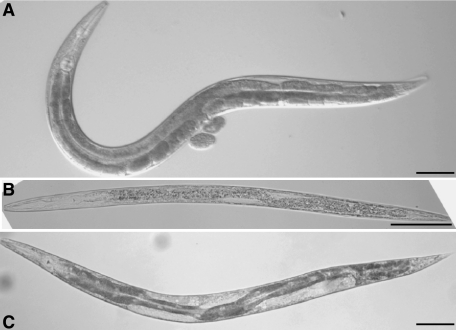
Developmental arrest in the dauer larval stage. Scale bar indicates 100 microns. (A) A wild-type adult hermaphrodite that is reproductively active and laying eggs. Under normal growth conditions, wild-type animals proceed through development to become fertile adults. (B) An *age-1(mg109)* animal arrested as a dauer larvae. Dauer larvae are morphologically distinct from reproductive adults and are nonreproductive. (C) A sterile *age-1(mg109)* adult. Under semipermissive conditions, some mutant animals can bypass dauer arrest, but form sterile, adult-sized animals lacking functional gonads (Gottlieb & Ruvkun, 1994).

Insulin and IGF-I are peptide hormones that signal through dimeric receptor tyrosine kinases to activate phosphoinositide 3-kinase (PI3K), resulting in the production of phosphoinositide-3,4,5-P_3_ (PI-3,4,5-P_3_) and phosphoinositide-3,4-P_2_ (PI-3,4-P_2_). Major effectors of the phospholipid products of PI3K are the serine/threonine kinases, AKT/PKB and PDK-1. PDK-1 phosphorylates and activates PIP-bound AKT/PKB, which, in turn, phosphorylates downstream target proteins (Fig. 1A). In *C. elegans*, all the components of this signaling pathway are conserved and function to modulate wild-type longevity, stress resistance and reproductive development. Mutations in *daf-2*, encoding an insulin receptor-like protein, or *age-1*, encoding the catalytic subunit of PI3K, increase adult longevity and stress resistance and can cause constitutive developmental arrest as dauer larvae, an alternative third stage larval form that is optimized for long-term survival under harsh environmental conditions (Fig. 2) (Kenyon *et al*., 1993; Malone *et al*., 1996; Morris *et al*., 1996; Kimura *et al*., 1997). In addition, LY294002, a chemical inhibitor of PI3 K, altered lifespan, stress resistance and dauer formation in *C. elegans* (Babar *et al*., 1999). Both IST-1, an IRS-like protein, and AAP-1, a p55-like PI3K adaptor protein, facilitate signaling between DAF-2/IR and AGE-1/PI3K (Wolkow *et al*., 2002). The phospholipid products of AAP-1/AGE-1 PI3K activate downstream serine/threonine kinases AKT-1 and PDK-1 (Paradis & Ruvkun, 1998; Paradis *et al*., 1999). The serum glucocorticoid kinase, SGK-1, also transduces signals from PI3K in both mammals and nematodes (Brunet *et al*., 2001; Hertweck *et al*., 2004).

In *C. elegans*, the major target of insulin-like signaling is the FOXO transcription factor, DAF-16, whose mammalian orthologs are FOXO1, FOXO3a and FOXO4 (Lin *et al*., 1997; Ogg *et al*., 1997). DAF-2/IR signaling activates AKT-1, which phosphorylates DAF-16, blocking DAF-16 nuclear entry (Henderson & Johnson, 2001; Lee *et al*., 2001; Lin *et al*., 2001). When signaling through the DAF-2 pathway is turned off, AKT-1-mediated inhibition of DAF-16 is relieved and DAF-16 translocates into the nucleus to promote target gene expression. It is believed that the direct transcriptional targets of DAF-16/FOXO are responsible for the stress resistance and longevity phenotypes associated with *daf-2*/IR mutations.

Several important studies examining the transcriptome of *daf-2(−)* strains have elucidated some tantalizing molecular clues about the mechanisms for insulin's effects on longevity. In these studies, gene expression in long-lived *daf-2(−)* strains was examined using full-genome microarrays or serial analysis of gene expression (SAGE) techniques (McElwee *et al*., 2003; Murphy *et al*., 2003; Halaschek-Wiener *et al*., 2005). In a complementary study, a biochemically defined DAF-16 binding site was used to define DAF-16 target genes by computational methods, focusing on *C. elegans* genes with orthologs in the *Drosophila* genome and conserved DAF-16 binding sites (Furuyama *et al*., 2000; Lee *et al*., 2003). Not surprisingly, given the high sensitivity of the expression analysis methods, the sets of affected transcripts did not fully overlap among the studies. However, similar changes were detected in specific genes, as pointed out by Halascheck-Wiener *et al*. (2005) (Table 1). In particular, general stress-response genes, such as heat-shock proteins, were up-regulated in *daf-2(−)* strains, while genes associated with reproduction and growth, such as vitellogenin yolk proteins, were down-regulated.

**Table 1:** Major distinguishing features of the *daf-2(−)* transcriptome

Functional class	Transcript (fold-up, -down)
	**UP in *daf*-*2(−)***
Stress resistance	*hsp-16*s and *hsp-12*s (1.8–253.4*†) (3.4–89‡)
	SOD (10.2–17.4‡)
	glutathione S-transferase (4–6‡)
Protein synthesis	Ribosomal subunit genes (*rpl-*, *rps-* genes, 2.4–2.8*†)
Signaling	Transthyretin genes (7.1*)
	Peptide neurotransmitter-like (8.2*) (3.4–4.1‡)
	7-TM receptor (5.1‡)
	Protein phosphorylation (3.6‡)
Gene expression	*tts-1* (21.2–65.3*†)
Collagens	*col* genes (3.6–16.4†)
	F02D10.1 (27.8‡)
Metabolism	*fat*-genes (5.1†)
Proteolysis	proteases (151–161‡)
	**DOWN in *daf*-*2(−)***
Fertility	vitellogenin yolk protein (39.3–385.1*) (17–33‡)
Stress response	*cey-1, -2, -3* (5.4–15.1*†)
Cell growth	Tubulin genes (30.1*)
	Actin genes (26.6*)
	RNA metabolism (*puf* genes, 5.8–19.6*†)
	DNA metabolism (*mcm* genes, 15.6–19.4*†)
	Protein translation (5.9–28.2*)
Proteolyis	*asp* genes, *spp*. genes, *rpn* genes (3.2–8.4*) *cct* genes (6.3*)

* Halaschek-Wiener *et al*. (2005): *daf-2(−)* vs. *daf-2(+)*, same ‘chronological’ age.

† Halaschek-Wiener *et al*. (2005): *daf-2(−)* vs. *daf-2(+)*, same ‘biological’ age.

‡ McElwee *et al*. (2003).

These examinations of the *daf-2(−)* transcriptome showed clearly that expression of stress-response genes was likely to be related to increased lifespan in these strains. Indeed, complementary studies have shown that changes of these genes can have direct effects on longevity. In particular, overexpression of *hsp-16* extended lifespan of wild-type and *age-1(−)* animals (Walker & Lithgow, 2003). In addition, dsRNA-mediated knockdown of the genes that were up-regulated in *daf-2(−)* animals was able to partially suppress the increased longevity phenotype (Lee *et al*., 2003; Murphy *et al*., 2003). The gene expression changes also appear to suggest that long lifespan may result, in part, from modifying resource allocation. For example, various DNA and protein metabolism genes were reduced in long-lived *daf-2(−)* animals (Table 1). Consistent with this hypothesis, lifespan of wild-type animals was increased by RNAi-mediated knockdown of genes down-regulated in *daf-2(−)*, suggesting that those gene activities incurred a cost to survival of the organism (Murphy *et al*., 2003)

Interestingly, transcriptome analyses identified both up- and down-regulated genes as putative DAF-16 targets and both types of transcriptional targets contain putative DAF-16 binding sites in their promoters (Murphy *et al*., 2003). It remains to be determined whether these are all direct DAF-16 target genes *in vivo*. If all are bona fide targets of DAF-16, this would suggest that DAF-16 can both increase and decrease gene expression under similar conditions. Perhaps this dual role is accomplished via interactions with partner proteins that assemble activator or repressor complexes at promoters. Alternatively, DAF-16 may directly affect expression of relatively few target genes that trigger a cascade of downstream gene expression changes. In either case, it will be exciting to decipher the details of these various effects.

## Identification of tissues where DAF-2/insulin signaling regulates longevity

The cellular effects of insulin and IGF-I in mammals are varied and complex, due to the fact that both peptides have endocrine, paracrine and autocrine effects on a variety of tissues. Insulin is produced mainly by pancreatic beta cells and IGF-I is produced by a wide variety of cells throughout the body, with hepatic IGF-I accounting for the majority of circulating bound IGF-I. In most tissues, insulin and IGF-I binding stimulate cell growth. Circulating glucose can stimulate insulin release, while many factors promote IGF-I secretion, including pituitary growth hormone (GH) and muscle contraction. Insulin binding to target cells stimulates glucose uptake and increases glucose metabolism, in part through effects on gene expression. IGF-I promotes growth and the absence of the IGF-I signaling system impairs growth during development (Butler & LeRoith, 2001). Although the liver contributes the majority of bound IGF-I in the circulation, liver-specific IGF-I knockouts revealed that hepatic IGF-I secretion is not essential for growth and suggested that growth-promoting IGF-I can be provided by other tissues (Yakar *et al*., 1999). In addition to these cellular effects, binding of insulin and IGF-I can affect the release of downstream factors, promoting secondary effects through the body.

Several recent reports have investigated the cell biological aspects of DAF-2 signaling in *C. elegans*. The *C. elegans* genome contains 38 *ins* genes that encode insulin-like peptides, some of which have been shown to act as either agonists or antagonists of the *daf-2* pathway (Pierce *et al*., 2001; Hua *et al*., 2003; Li *et al*., 2003). The *C. elegans ins* genes are expressed throughout the body, with most showing some kind of neuronal expression (Pierce *et al*., 2001). This widespread *ins* expression may indicate that these peptides can act locally, through paracrine or autocrine effects, as opposed to endocrine effects. In *C. elegans*, relatively little is known about the factors that stimulate INS release. One factor may be from environmental stimuli detected by sensory neurons, as mutations disrupting sensory neuron function can increase lifespan, although INS production has not been directly examined in these animals (Apfeld & Kenyon, 1999; Alcedo & Kenyon, 2004). The gonad may also contribute signals promoting INS release. The removal of the germ line, either through genetic mutation or laser ablation of germ cell precursors, increases lifespan and this effect appears to occur via interactions with *daf-16*/FOXO activity in the intestinal cells (Arantes-Oliveira *et al*., 2002; Libina *et al*., 2003). One possible mechanism to explain this set of observations is that gonad signals affect activity of one or several *ins* genes that bind to DAF-2 receptors in the intestinal cells.

Binding of INS peptide ligands activates signaling through the DAF-2 receptor. Several studies have set out to determine whether DAF-2/DAF-16 signaling promotes wild type lifespan from specific cells or sets of cells. Mosaic and transgenic analysis showed that DAF-2 and AGE-1 could function non-cell autonomously from either the nervous system or intestine to promote wild-type lifespan (Apfeld & Kenyon, 1998; Wolkow *et al*., 2000). Since DAF-16 is a direct target of AKT-1, which is downstream of DAF-2 and AGE-1, it seemed likely that DAF-16 would function in the same cells as AGE-1 and DAF-2. It was therefore surprising to find that mosaic and transgenic analysis indicated that *daf-16* function in the intestine was necessary for increased lifespan in *daf-2(−)* animals, while neuronal *daf-16* activity only promoted dauer arrest (Libina *et al*., 2003). Taken together, these reports appear to suggest that DAF-2 signaling need not function in the same cells as DAF-16/FOXO. In *Drosophila*, lifespan was lengthened by overexpressing *Drosophila* FOXO in fat bodies, but not neurons, seeming to support the *C. elegans* results with *daf-16* (Giannakou *et al*., 2004; Hwangbo *et al*., 2004).

One possible explanation for the apparent inconsistency in the cells where DAF-2 and DAF-16 seem to function is that DAF-2 signaling may affect DAF-16/FOXO both cell autonomously, through AKT-1 activity, as well as non-cell autonomously, through downstream pathways. One possible pathway is feedback regulation via the insulin-like (INS) ligands, which are expressed throughout the body, provided that the mutant backgrounds utilized to test this idea could respond, at least partially, to INS ligands. Alternatively, DAF-2 signaling could impact downstream pathways that, in turn, transmit DAF-2 signaling throughout the body. A third possibility is that DAF-16 activity may be regulated by pathways that function in parallel to AKT-1.

## Pathways that interact with insulin signaling and control longevity

A new player that is now known to participate in the DAF-16/FOXO regulation in *C. elegans* and *Drosophila* is the Jun N-terminal kinase (JNK) (Fig. 1B) (Oh *et al*., 2005; Wang *et al*., 2005). In *C. elegans*, activation of JNK signaling, either by overexpression or by genetic relief of inhibitory inputs, promoted DAF-16/FOXO nuclear translocation and increased lifespan. In *Drosophila*, JNK activation also mediated feedback inhibition of insulin-like peptide secretion, further attenuating insulin receptor signaling (Wang *et al*., 2005). In *C. elegans*, a separate p38 MAP kinase, SEK-1, may also promote DAF-16 nuclear translocation during oxidative stress (Kondo *et al*., 2005). These studies indicate that DAF-16 can be affected by signaling through at least one kinase cascade apparently independently of DAF-2.

Another gene that can increase lifespan through interactions with DAF-16 is heat-shock factor-1 (HSF-1) (Fig. 1B) (Hsu *et al*., 2003; Morley & Morimoto, 2004). *hsf-1* activation by stress promotes expression of inducible heat-shock proteins (*hsps*). The observation that *hsp*s are targets of DAF-16 suggested the possibility that DAF-16 and HSF-1 could collaborate at promoters. Indeed, sequence and genetic analysis supported this idea. DAF-16 is also assisted by the β-catenin, BAR-1, which is required for oxidative stress resistance and expression of antioxidant gene targets of DAF-16 (Fig. 1B) (Essers *et al*., 2005).

Nutrient availability affects longevity through two pathways that interact with the *daf-2* pathway (Fig. 1C). The histone deacetylase, SIRT1, which is a major regulator of the cellular response to caloric restriction, also modulates the activity of DAF-16 and promotes longer lifespan (Tissenbaum & Guarente, 2001; Wood *et al*., 2004). In addition, the mammalian ortholog, SIRT1, can deacetylate FOXO3a, thereby altering the transcriptional activation of FOXO3a target genes (Brunet *et al*., 2004; Motta *et al*., 2004). As SIRT1 activity is responsive to cellular metabolic status, the effect of SIRT1 on FOXO activity provides a way for cellular metabolism to link to FOXO target genes, which may include stress resistance and metabolism genes, as they do in *C. elegans*.

The *daf-15* gene, encoding the RAPTOR-like subunit of the metabolic regulator, TOR, is a downstream target of DAF-16 in the dauer arrest pathway (Jia *et al*., 2004). Mutations in *daf-15* cause larvae to arrest development as nonrecovering dauer-like larvae. In addition, adult lifespan was increased in animals with weak *daf-15* mutations that could bypass larval arrest. These phenotypes are similar to those observed for animals with mutations in *CeTOR*, encoded by the gene, *let-363*, indicating that DAF-15/RAPTOR and CeTOR act together to transduce nutrient signals (Vellai *et al*., 2003). Genetic analysis showed that *daf-15* likely acts downstream of *daf-16* for dauer arrest and *daf-16* activity was dispensible for increased lifespan in *CeTOR/let363(−)* adults (Fig. 1C) (Vellai *et al*., 2003). Furthermore, transcriptional analysis revealed that *daf-15* expression was significantly decreased in long-lived *daf-2(e1370)* animals (Jia *et al*., 2004). These findings show that the TOR nutrient-sensing pathway acts in parallel, and downstream of, insulin signaling to regulate larval development and adult longevity.

Another gene that deserves to be noted in this discussion of interacting pathways is *old-1*, which encodes a predicted tyrosine kinase. *old-1* was identified in a screen for genes that increased longevity when overexpressed, an unusual approach in *C. elegans*, where genetic screens are usually performed by mutating the genome (Murakami & Johnson, 2001). Interestingly, *old-1* mRNA levels were also increased in long-lived *daf-2(−)* animals, suggesting this protein may be important for longevity in these animals (Murphy *et al*., 2003). Clearly, more remains to be learned about OLD-1's role in determining lifespan.

## Activating mutations in the DAF-2 pathway suggest targets for pharmacological intervention

The strong developmental phenotypes of mutations inactivating DAF-2 pathway signaling facilitate genetic identification of mutations that affect lifespan. In the absence of DAF-2 pathway signaling, animals constitutively arrest development at the dauer larval stage. Normally, *C. elegans* embryos develop through four larval stages (L1–L4), differing anatomically and by size, before molting into reproductive adults. First stage (L1) larvae may opt to enter an alternative third larval stage, called the dauer, in the presence of environmental conditions that are suboptimal for reproduction, such as limited nutrients, high population density or high temperature (Riddle & Albert, 1997). As dauer larvae, animals can arrest development and survive for several months until environmental conditions improve. Morphological features that enhance long-term survival in dauer larvae include thickening of the cuticle and closure of the bucchal and anal openings to resist dessication, fat storage for metabolic needs, and increased expression of antioxidant enzymes, such as SOD and HSPs (Fig. 2).

Mutations that severely disrupt DAF-2 pathway signaling cause larvae to arrest development at the dauer larval stage, regardless of environmental conditions (Gottlieb & Ruvkun, 1994; Malone *et al*., 1996). In addition, weak decrements in signaling through the DAF-2 pathway can cause animals to develop into sterile adult-sized animals lacking a functional gonad (Gems *et al*., 1998) (Fig. 2). Under some conditions, dauer arrest is a terminal phenotype and animals cannot recover into reproductive adults. This feature of the *daf-2* pathway mutants suggests that this pathway also has important functions in dauer recovery. Developmental arrest of *daf-2* pathway mutants facilitates the straightforward identification of alleles that can activate signaling through this pathway. In these screens, suppressor alleles are selected that allow *daf-2* pathway mutants to bypass dauer arrest and complete reproductive development. Due to the tight dauer arrest phenotype of the starting strain, this screening strategy allows the rapid examination of large numbers of mutagenized animals for rare reproductive adults.

Screening for suppressors of dauer arrest in *age-1(−)* animals led to the identification of activating mutations in the downstream kinases, AKT-1 and PDK-1 (Paradis & Ruvkun, 1998; Paradis *et al*., 1999). The activating mutation in AKT-1 was an Ala183Thr mutation in the linker region between the phospholipid-binding PH domain and the kinase domain (Paradis & Ruvkun, 1998). One scenario for how Ala183Thr activates AKT-1 is by disrupting autoinhibition of AKT-1 and increasing the basal activity level of the mutant protein. Although the linker regions of the human and worm orthologs differ in sequence, they are likely to carry out similar functions. Thus, it might be possible to engineer compounds that could mimic the effect of the Ala183Thr mutation to activate AKT signaling. This type of drug would be beneficial for treating diseases where AKT activity is limited, such as for restoring insulin sensitivity in the context of diabetes mellitus. Indeed LY294002 has been shown to mimic phenotypes seen in AGE-1/PI3K mutant worms highlighting how drugs can be useful in pharmacologically manipulating insulin-like signaling in the worm (Babar *et al*., 1999). The ease of screening for suppressors of the *daf-2(−)* dauer arrest phenotype will facilitate the future identification of additional mutations that activate AKT and PDK-1 kinases.

## Concluding remarks

The goal of developing drugs for manipulating lifespan is an ambitious one. The chances of success in this endeavor will increase if the proteins that are ultimately the best drug targets are identified early in the discovery process. Studies of short-lived invertebrates offer the best possibility to rapidly identify and characterize pathways that have strong effects on longevity. Further characterization and validation of these pathways in mammals will follow with the development of assays for studying aging within various tissues. The insulin pathway confers the most dramatic effects to date on longevity in *C. elegans* and *Drosophila*. For this reason, it will be an obvious target for manipulating longevity in mammals. Furthermore, the importance of insulin and IGF-I signaling in human diseases, such as diabetes and cancer, ensure that each new finding will have important ramifications for human health, notably that of the elderly.
